# Acute Myeloid Leukemia Evolving from JAK 2-Positive Primary Myelofibrosis and Concomitant CD5-Negative Mantle Cell Lymphoma: A Case Report and Review of the Literature

**DOI:** 10.1155/2012/875039

**Published:** 2012-08-02

**Authors:** Diana O. Treaba, Salwa Khedr, Shamlal Mangray, Cynthia Jackson, Jorge J. Castillo, Eric S. Winer

**Affiliations:** ^1^Department of Pathology and Laboratory Medicine, Rhode Island Hospital, The Warren Alpert Medical School, Brown University, Providence, RI 02903, USA; ^2^Division of Hematology/Oncology, The Miriam Hospital, The Warren Alpert Medical School, Brown University, Providence, RI 02904, USA

## Abstract

Primary myelofibrosis (formerly known as chronic idiopathic myelofibrosis), has the lowest incidence amongst the chronic myeloproliferative neoplasms and is characterized by a rather short median survival and a risk of progression to acute myeloid leukemia (AML) noted in a small subset of the cases, usually as a terminal event. As observed with other chronic myeloproliferative neoplasms, the bone marrow biopsy may harbor small lymphoid aggregates, often assumed reactive in nature. In our paper, we present a 70-year-old Caucasian male who was diagnosed with primary myelofibrosis, and after 8 years of followup and therapy developed an AML. The small lymphoid aggregates noted in his bone marrow were neoplastic in nature and represented bone marrow involvement by a CD5-negative mantle cell lymphoma (MCL) that presented without any associated lymphadenopathy. We reviewed the English medical literature to identify a single case report of simultaneous association of AML and a MCL in the bone marrow. The unusual association presented here suggests an increase in observer awareness to apparently benign lymphoid aggregates in chronic myeloproliferative neoplasms.

##  Discussion

**Figure 1:**
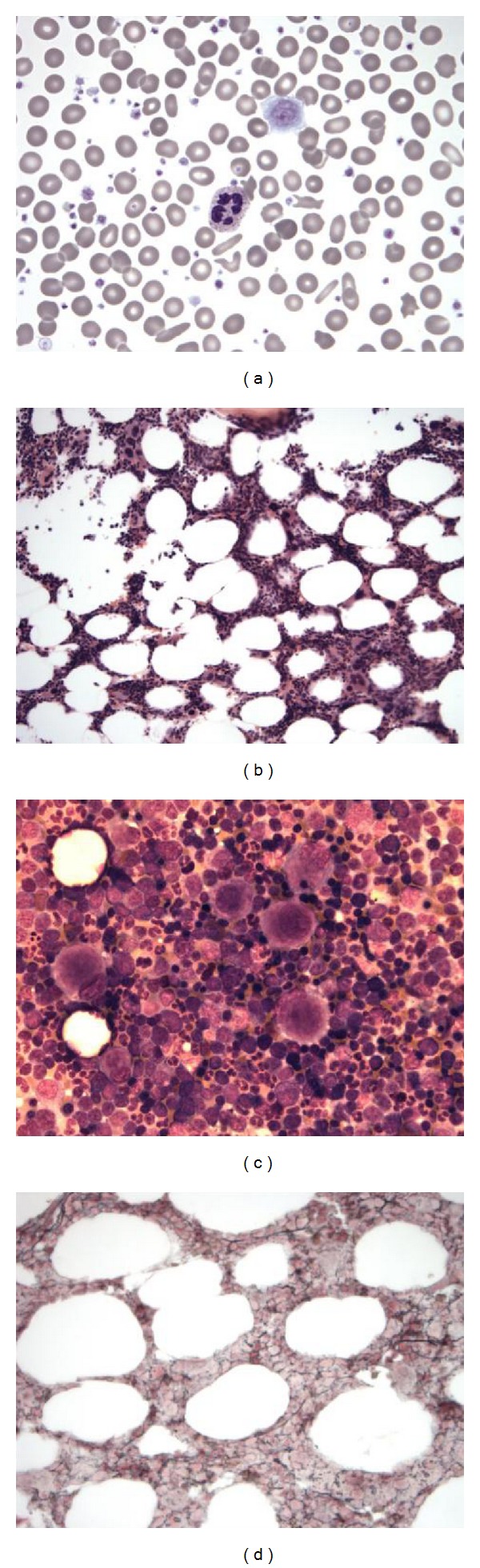
Primary myelofibrosis, prefibrotic stage: (a) peripheral blood, thrombocytosis with giant platelets, Wright-Giemsa stain, Ob. 100x, immersion oil. (b) bone marrow biopsy with mildly hypercellular bone marrow, hematoxylin and eosin stain, Ob. 40x. (c) bone marrow aspirate, dysmegakaryopoiesis, Wright Giemsa stain, Ob. 100x, immersion oil. (d) Reticulin stain, mildly increased reticulin deposition. Ob. 50x.

A 70-year-old male patient with a past medical history of left hip replacement and depression presented with arthralgias involving the neck, hips, and knees. The complete blood count (CBC) had significant changes from normal indices noted on a previous examination performed 2 months earlier and showed leukocytosis (White blood cell (WBC) count 16.7 × 10^9^/L; normal range 3.5–11.0 × 10^9^/L) with neutrophilia (absolute neutrophil count 13.2 × 10^9^/L; normal range 1.5–7.5 × 10^9^/L) and thrombocytosis of 1,346 × 10^9^/L (normal range 150–400 × 10^9^/L)). The white blood cell differential included segmented neutrophils 78%, monocytes 5%, lymphocytes 11%, and basophils 6%. His red blood cell (RBC) count was 4.72 × 10^12^/L (normal range 4.2–5.5 × 10^12^/L), hemoglobin 13.8 g/dL (normal range 13.5–16.0 g/dL), and the RDW was 15.9% (normal range 11.5–14.5%) (Table 1). The peripheral blood smear showed neutrophilia with a subset of hypersegmented neutrophils, (RBCs) with moderate anisopoikilocytosis, and also thrombocytosis with a subset of giant platelets (Figure 1(a)). Physical examination did not reveal hepatosplenomegaly or lymphadenopathy. The bone marrow was mildly hypercellular (50%) (Figure 1(b)), had trilineage hematopoiesis, a normal M : E ratio, megakaryocytic hyperplasia (9.5 megakaryocytes/hpf of 40x), and dysmegakaryopoiesis (Figure 1(c)). The iron stores were normal, and sideroblasts were not identified. A reticulin stain was remarkable for a mildly increased (2+/4) deposition of reticulin fibers (Figure 1(d)). The cytogenetic analysis showed a normal karyotype (46, XY). Other laboratory values were significant for an elevated lactate dehydrogenase (LDH), 251 IU/L (normal: 50–175 IU/L). A chronic myeloproliferative neoplasm was highly suspected, and the differential diagnosis included essential thrombocythemia, the early onset of primary myelofibrosis, and also a possible myelodysplastic/myeloproliferative neoplasm. Therapy with anagrelide (2 × 0.5 g/day) was started and progressively increased, reaching a stable dose of 3 g/day to maintain the platelet count below 600 × 10^9^/L.

**Figure 2:**
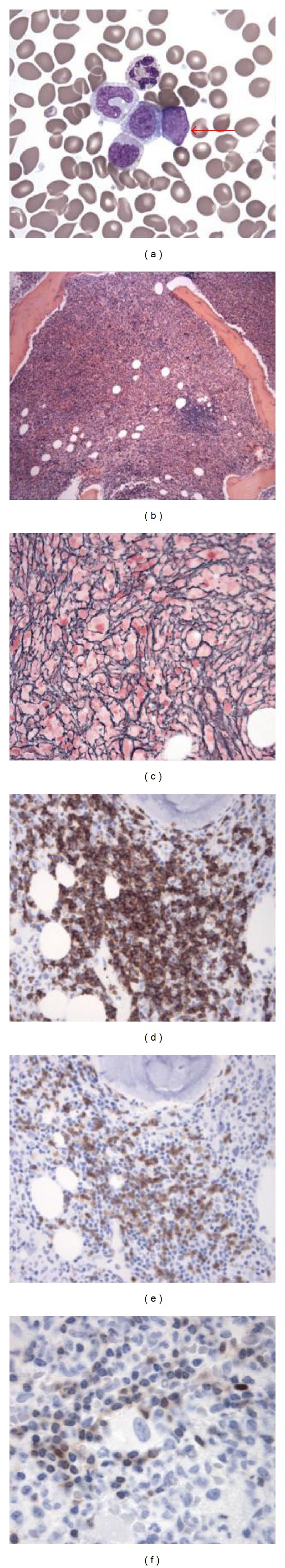
Primary myelofibrosis, fibrotic stage: (a) peripheral blood smear, Wright-Giemsa stain, blast (arrow), Ob. 100x immersion oil. (b) Hypercellular bone marrow biopsy, Hematoxylin and eosin stain, Ob. 50x (c) bone marrow biopsy with markedly increased reticulin deposition. Ob. 50x, (d) Anti-CD20 antibody highlights a large subset of positive lymphoid cells in a lymphoid aggregate, Ob. 50x. (e) Anti-CD3 antibody highlights a small subset of CD3+ T-cells in a lymphoid aggregate, Ob. 50x. (f) Anti-Cyclin D1 antibody highlights a small subset of positive nuclei in a lymphoid aggregate, Ob. 50x.

Two years later the patient had a cerebrovascular accident. A second bone marrow examination showed increased cellularity, megakaryocytic hyperplasia, dysmegakaryopoiesis, and marked fibrosis (4+/4). A small intertrabecular lymphoid aggregate composed of small mature appearing lymphocytes was also noted and favored to be reactive in nature. The CBC and peripheral blood smear were similar to those noted in 2003 except for anemia; lymphadenopathy was not observed and a CT scan of the abdomen detected mild splenomegaly. Therapy was switched to hydroxyurea, then restarted and titrated to maintain platelet counts in the range of 450–550 × 10^9^/L. A PCR analysis performed on a blood sample detected the presence of the JAK 2-V617 mutation. Notable changes in the CBC and peripheral blood smear morphology were detected in August 2010 when the WBC reached 59.3 × 10^9^/L, and a leukoerythroblastic picture was noted (Figure 2(a)) with a white blood differential including segmented neutrophils 80%, bands 6%, lymphocytes 2%, monocytes 3%, eosinophils 1%, basophils 1%, meta/myelocytes 2%, and blasts 5%. A subset of neutrophils had hypersegmented nuclei; a few hypogranular neutrophils were also noted and there was associated macrocytic anemia (hemoglobin 12.6 g/dL, MCV: 113.1 fL) and tear drop RBCs. The platelets remained in the normal range (303 × 10^12^/L). The bone marrow aspirate sampled was a “dry tap,” but the biopsy was markedly hypercellular (cellularity 95%) with megakaryocytic hyperplasia (>10 megakaryocytes/hpf) and showed dysmegakaryopoiesis, a few small ill-defined intertrabecular lymphoid aggregates (Figure 2(b)) and also marked fibrosis (4+/4) (Figure 2(c)). By immunohistochemistry, there were approximately 9% CD34-positive blasts and the small lymphoid aggregates consisted of a larger population of CD20+ B-lymphoid cells (Figure 2(d)) than CD3+ T cells (Figure 2(e)). The B cells were positive for bcl2 and negative for CD5 and CD10. Few scattered cyclin D1+ nuclei were noted in some of these lymphoid aggregates, raising the suspicion of mantle cell lymphoma (Figure 2(f)). Molecular studies detected the presence of immunoglobulin heavy chain gene rearrangements and were negative for IgH-bcl2 and IgH-bcl1 translocations. Therapy with hydroxyurea was increased at 2 g/day and the WBC count decreased below 20 × 10^9^/L in the next months.

**Figure 3:**
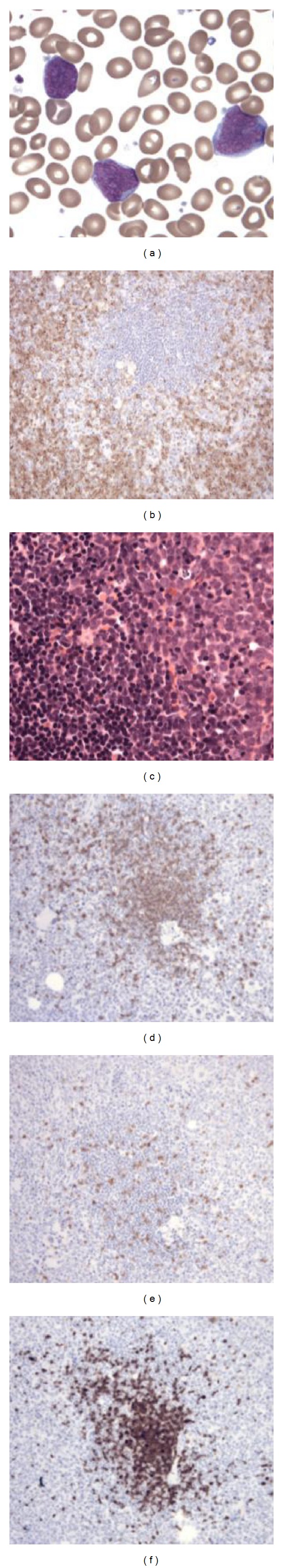
Acute myeloid leukemia and associated bone marrow involvement by mantle cell lymphoma: (a) peripheral blood smear, Wright-Giemsa stain, blasts, Ob. 100x, immersion oil. (b) Bone marrow biopsy, Ob. 20x, anti-CD34 antibody highlights positive blasts; the lymphoid cells are negative. (c) Bone marrow biopsy, hematoxylin and eosin stain, Ob. 50x, lymphoid aggregate and blasts. (d) Bone marrow biopsy, anti-CD20 antibody highlights a large subset of positive cells in a lymphoid aggregate, Ob. 20x. (e) Bone marrow biopsy, anti-CD3 antibody highlights a small subset of positive cells in a lymphoid aggregate, Ob. 20x. (f) Bone marrow biopsy, anti-Cyclin D1 antibody highlights a large subset of positive cells in a lymphoid aggregate, Ob. 20x.

In 2011, while presenting with symptoms suggestive of a respiratory infection, the CBC showed leukocytosis (WBC 40.3 × 10^9^/L), hemoglobin of 11 g/dL, MCV 115 fL, platelets 215 × 10^12^/L (Table 1), and the peripheral blood smear was remarkable for 69% blasts (Figure 3(a)). The blasts were positive for myeloperoxidase cytochemical stain and by flow cytometry were positive for CD13, CD33, CD117, CD45, HLA-DR, CD34, CD11c, CD71, and CD11b. Bone marrow aspirate smears showed 73.4% blasts, markedly decreased maturing myeloid cells, dysgranulopoiesis, decreased erythroid series with mild dyserythropoiesis, and dysmegakaryopoiesis. The hypercellular bone marrow biopsy (95%) had approximately 70% CD34+ blasts (Figure 3(b)) and several small-to-medium size lymphoid aggregates (Figure 3(c)), consisting of small to medium sized lymphoid cells. The lymphoid aggregates involved approximately 10% of the bone marrow space and consisted of CD20+ (Figure 3(d)), bcl2+, CD5-negative, bcl6-negative B-lymphoid cells, admixed with a small subset of CD3+ T-cells (Figure 3(e)). The B-lymphoid cells had variable expression of cyclin D1 (Figure 3(f)) and a low proliferation rate (approx. 5% to focally 10–20%). Molecular studies detected the presence of immunoglobulin heavy chain gene rearrangements, and a FISH analysis showed a low level of positivity for CCND1/IGH fusion and gain of IgH. Cytogenetics studies revealed a normal male karyotype and a PCR study was negative for the FLT3 mutation. A diagnosis of secondary acute myeloid leukemia (AML) evolving from primary myelofibrosis (PMF) and concomitant CD5-negative mantle cell lymphoma (MCL) was rendered. Patient's physical and CT examination was remarkable for hepatosplenomegaly without lymphadenopathy. The patient was started on a 10-day regimen with decitabine, which was stopped at day 9 due to his progressively altered mental status, and soon after the patient expired.

PMF is a Philadelphia chromosome-negative chronic myeloproliferative neoplasm characterized by a clonal proliferation of hematopoietic cells with variable morphologic maturity and efficiency [3–5]. The disease has a gradual progression from a hypercellular bone marrow with hyperplastic granulopoiesis and atypical megakaryocytes to a fibrotic bone marrow, extramedullary hematopoiesis, leukoerythroblastic picture, tear drop RBCs, and associated hepatosplenomegaly [3]. PMF is an uncommon disease with an estimated incidence in United States ranging from 0.5 to 1.5 per 100,000 persons per year [3, 6] and occurs mainly in middle-aged and elderly patients, with a median age at presentation of 67 years [6]. Approximately 5 and 17% of the patients are diagnosed before the age of 40 and 50, respectively [7]. The disease is rare in childhood, may be familial or idiopathic, and may be associated with congenital anomalies and chromosome abnormalities [8, 9]. A Janus-associated kinase 2 (JAK 2) gene mutation is identified in approximately 35–57% of the patients with PMF [10]. PMF carries a risk of transformation to AML that ranges from 8% to 23%, which is not necessarily associated with a previous therapy [11, 12]. Patients with JAK 2-V617F mutation-positive PMF have a higher risk of leukemic transformation [13, 14]. The majority of leukemic transformations have been of myeloid origin (includes all French-American-British classification subtypes) [15–17]; however, lymphoblastic [18] and mixed lineage leukemias [19] have also occasionally been described. A poor response to chemotherapy is usually noted, and in the series of 91 patients reported by Mesa et al., the treatment with AML-like induction therapy yielded a complete remission rate of zero and a treatment-related mortality of 33% [15].

A recent study evaluating 1000 patients verified a prognostic scoring system for these patients as well as provided valuable clinical and demographic information [20].

 Mantle cell lymphoma (MCL) is a mature, aggressive B-cell lymphoma with a CCND1 translocation, usually presenting at an advanced clinical stage, and commonly involving the lymph nodes, spleen, bone marrow, and extranodal sites (includes the gastrointestinal tract and Waldeyer ring) [19]. It occurs in middle-aged to elderly patients and has a male predominance. The neoplastic B-lymphoid cells are CD5 and CD43 positive and usually negative for CD10, bcl6, and CD23. Cases of CD5-negative MCL have been previously reported [21, 22] and appear to comprise approximately 11% of the cases of MCL [22]. While their detailed characterization is still to be completed, data from a small series of 7 cases suggests that these cases have similar clinical features to their CD5-positive counterparts, but may be associated with an unusually long survival, noted in 3 out of 7 cases described [21]. In two separate case report studies, CD5-negative MCL remarkable for splenic involvement and lacked lymphadenopathy features similar to our case [23, 24].

The unusual simultaneous association of an AML and MCL has been previously described in a single case report [25], and the AML (an acute monoblastic leukemia) was secondary to prior chemotherapy for breast carcinoma. Hsieh et al. described a second case where a temporal connection between the two diseases is noted in a 52-year-old male patient who developed AML 3 years after chemotherapy for his MCL. However, in this second case, there wasnot a proven concomitant bone marrow involvement by both lymphoma and leukemia [26].

Our case highlights the rare occurrence in a bone marrow replaced by JAK 2-positive PMF of an unusual variant of MCL, CD5 negative, and also the later “coexistence” of the MCL with an AML. While the association noted here might be merely coincidental, it brings attention to the mechanisms of a B-cell clone expansion in a clearly fibrotic marrow and to the underlying cell-to-cell interactions. Of note, this CD5-negative mantle cell lymphoma had a lowproliferation rate and was not associated with lymphadenopathy on repeated imaging studies. While a few prior studies [23, 24] suggested that this variant of MCL may have a more indolent behavior, it is not clear if it had or not an impact on the overall progression of the primary myelofibrosis or in the leukemic transformation.

Myelofibrosis has been described in association with non-Hodgkin lymphomas [27], and the stromal fibrosis in these cases is suggested to develop secondary to TGF-beta secreted by the lymphoma cells. However, in the cases analyzed an associated JAK 2-V617 mutation could not be demonstrated as was in our case.

While benign lymphoid aggregates have been described in bone marrow biopsies in association with chronic myeloproliferative disorders [26], the current case raises the awareness for immunophenotypical evaluation of the apparently benign lymphoid aggregates present in the bone marrow.

## Figures and Tables

**Table 1:** Sequential analysis of peripheral blood and bone marrow changes.

	Diagnosis			
	Prefibrotic stage of primary myelofibrosis	JAK 2-positive primary myelofibrosis (IPSS score 1; DIPSS score 1 [1, 2])	JAK 2-positive primary myelofibrosis and atypical B-lymphoid aggregates (IPSS score 3, DIPSS score 4)	Acute myeloid leukemia and CD5-negative mantle cell lymphoma
Time elapsed	Onset	2 years	7 years	7 years and 9 months

Peripheral Blood				
WBC (×10^9^/L)	16.7	16.4	59.3	40.3
Hgb (g/dL)	13.8	10.1	12.6	11
Platelets (×10^9^/L)	1346	575	303	202
Blasts (%)	0	0	5%	69%

Bone Marrow				
Cellularity	50%	95%	95%	95%
Reticulin	2+/4	4+/4	4+/4	4+/4
Blasts (%)	<5%	<5%	9%	73.4%

Description	Megakaryocytic hyperplasia, dysmegakaryopoiesis	Dysmegakaryopoiesis	Dysmegakaryopoiesis	Blasts, dysmegakaryopoiesis

Lymphoid aggregates	None	Single small aggregate Cyclin D1 negative IgH negative	CD20+, CD5 negative, BCL2+, A few cells are Cyclin D1+ IgH positive	Lymphoid aggregates 10%, CD5 negative, CD20+, BCL2+ Cyclin D1+ IgH positive FISH positive for t(11;14)

## References

[1] Cervantes F, Dupriez B, Pereira A (2009). New prognostic scoring system for primary myelofibrosis based on a study of the International working group for myelofibrosis research and treatment. *Blood*.

[2] Passamonti F, Cervantes F, Vannucchi AM (2010). A dynamic prognostic model to predict survival in primary myelofibrosis: a study by the IWG-MRT (International Working Group for Myeloproliferative Neoplasms Research and Treatment). *Blood*.

[3] Thiele J, Kvasnicka HM, Tefferi A, Swerdlow SH, Campo E, Harris NL (2008). Primary myelofibrosis. *WHO Classification of Tumors of Haematopoietic and Lymphoid Tissues*.

[4] Mesa RA, Verstovsek S, Cervantes F (2007). Primary myelofibrosis (PMF), post polycythemia vera myelofibrosis (post-PV MF), post essential thrombocythemia myelofibrosis (post-ET MF), blast phase PMF (PMF-BP): consensus on terminology by the international working group for myelofibrosis research and treatment (IWG-MRT). *Leukemia Research*.

[5] Tefferi A (2000). Myelofibrosis with myeloid metaplasia. *New England Journal of Medicine*.

[6] Mesa RA, Silverstein MN, Jacobsen SJ, Wollan PC, Tefferi A (1999). Population-based incidence and survival figures in essential thrombocythemia and agnogenic myeloid metaplasia: an olmsted county study, 1976–1995. *American Journal of Hematology*.

[7] Visani G, Finelli C, Castelli U (1990). Myelofibrosis with myeloid metaplasia: clinical and haematological parameters predicting survival in a series of 133 patients. *British Journal of Haematology*.

[8] Mallouh AA, Sa’di AR (1992). Agnogenic myeloid metaplasia in children. *American Journal of Diseases of Children*.

[9] Bonduel M, Sciuccati G, Torres AF, Pierini A, Gallo G (1998). Familial idiopathic myelofibrosis and multiple hemangiomas. *American Journal of Hematology*.

[10] Ahmed A, Chang CC (2006). Chronic idiopathic myelofibrosis: clinicopathologic features, pathogenesis, and prognosis. *Archives of Pathology and Laboratory Medicine*.

[11] Okamura T, Kinukawa N, Niho Y, Mizoguchi H (2001). Primary chronic myelofibrosis: clinical and prognostic evaluation in 336 japanese patients. *International Journal of Hematology*.

[12] Cervantes F, Tassies D, Salgado C, Rovira M, Pereira A, Rozman C (1991). Acute transformation in nonleukemic chronic myeloproliferative disorders: actuarial probability and main characteristics in a series of 218 patients. *Acta Haematologica*.

[13] Campbell PJ, Griesshammer M, Döhner K (2006). V617F mutation in JAK 2 is associated with poorer survival in idiopathic myelofibrosis. *Blood*.

[14] Barosi G, Bergamaschi G, Marchetti M (2007). JAK 2 V617F mutational status predicts progression to large splenomegaly and leukemic transformation in primary myelofibrosis. *Blood*.

[15] Mesa RA, Li CY, Ketterling RP, Schroeder GS, Knudson RA, Tefferi A (2005). Leukemic transformation in myelofibrosis with myeloid metaplasia: a single-institution experience with 91 cases. *Blood*.

[16] Hernandez JM, San Miguel JF, Gonzalez M (1992). Development of acute leukaemia after idiopathic myelofibrosis. *Journal of Clinical Pathology*.

[17] Batlle M, Fernandez-Aviles F, Ribera JM (1999). Acute promyelocytic leukemia in a patient with idiopathic myelofibrosis. *Leukemia*.

[18] Shaheen SP, Talwalkar SS, Simons R, Yam L (2005). Acute lymphoblastic leukemic transformation in a patient with chronic idiopathic myelofibrosis and paroxysmal nocturnal hemoglobinuria: a case report and review of the literature. *Archives of Pathology and Laboratory Medicine*.

[19] Swerdlow SH, Campo E, Seto M, Muller-Hermelink HK, Swerdlow SH, Campo E, Harris NL (2008). Mantle cell lymphoma. *WHO Classification of Tumors of Haematopoietic and Lymphoid Tissues*.

[20] Tefferi A, Lasho TL, Jimma T (2012). One thousand patients with primary myelofibrosis: the Mayo Clinic experience. *Mayo Clinic Proceedings*.

[21] Kaptain S, Zukerberg LR, Ferry JA (1998). Bcl-1/cyclin D1+ CD5- mantle cell lymphoma. *Modern Pathology*.

[22] Bell ND, King JAC, Kusyk C, Nelson BP, Sendelbach KM (1998). CD5 negative diffuse mantle cell lymphoma with splenomegaly and bone marrow involvement. *Southern Medical Journal*.

[23] Yoo SB, Kim YA, Jeon YK, Kim CW (2008). CD5-undetected by immunohistochemistry, t(11;14)(q13;q32)-positive conjunctival mantle cell lymphoma: a case report. *Pathology Research and Practice*.

[24] Carulli G, Marini A, Ciancia EM (2011). Discordant lymphoma consisting of splenic mantle cell lymphoma and marginal zone lymphoma involving the one marrow and peripheral blood: a case report. *Journal of Medical Case Reports*.

[25] Pawarode A, Baer MR, Padmanabhan S (2005). Simultaneous presentation of acute monoblastic leukemia and mantle cell lymphoma: case report and review of the literature. *Leukemia and Lymphoma*.

[26] Hsieh YC, Lin CL, Tsao CJ, Hsieh PP, Tzeng CC, Chuang SS (2009). Aberrant expression of CD19 and CD43 in a patient with therapy-related acute myeloid leukemia and a history of mantle cell lymphoma. *Kaohsiung Journal of Medical Sciences*.

[27] Etienne A, Gruson B, Chatelain D (2009). Myelofibrosis-associated lymphoproliferative disease: retrospective study of 16 cases and literature review. *Advances in Hematology*.

